# Library instruction and Wikipedia: investigating students' perceived information literacy, lifelong learning, and social responsibility through Wikipedia editing

**DOI:** 10.5195/jmla.2022.1291

**Published:** 2022-04-01

**Authors:** Melissa K. Kahili-Heede, Uday Patil, K. J. Hillgren, Earl Hishinuma, Richard Kasuya

**Affiliations:** 1 mkahili@hawaii.edu, Information Services and Instruction Librarian, Health Sciences Library, John A. Burns School of Medicine at the University of Hawai‘i at Mānoa, Honolulu, HI; 2 uday@hawaii.edu, Thompson School of Social Work and Public Health, University of Hawai‘i at Mānoa, Honolulu, HI; 3 hillgren@hawaii.edu, Health Sciences Library, John A. Burns School of Medicine at the University of Hawai‘i at Mānoa, Honolulu, HI; 4 hishinumae@dop.hawaii.edu, Department of Psychiatry, John A. Burns School of Medicine at the University of Hawai‘i at Mānoa, Honolulu, HI; 5 kasuya@hawaii.edu, Office of Medical Education, John A. Burns School of Medicine at the University of Hawai‘i at Mānoa, Honolulu, HI

**Keywords:** information literacy, social responsibility, Wikipedia

## Abstract

**Objectives:**

This article presents a multiyear pilot study delineating practical challenges, solutions, and lessons learned from Wikipedia editing experiences with first-year medical students at the John A. Burns School of Medicine at the University of Hawai‘i at Mānoa. The purpose of our project was to determine the feasibility and effectiveness of Wikipedia editing to improve information literacy and lifelong learning skills and to investigate aspects of social responsibility in first-year medical students.

**Methods:**

Lessons were provided through a combination of in-person and online instruction via the WikiEdu learning management system (LMS). Students next selected a health-related Wikipedia article to edit. After the editing experience, structural completeness data were collected from the WikiEdu LMS. Feedback was collected via an anonymous retrospective pre-post survey to assess the students' attitudes toward their perceived information literacy skills and the social responsibility of improving Wikipedia articles. Nonparametric tests were conducted to compare pre versus post outcomes.

**Results:**

Fifty-seven (79%) participants in the 2018 cohort and forty-nine (64%) participants in the 2019 cohort completed the retrospective pre-post survey. In both cohorts, respondents showed statistically significant increases (*p*<.05) in self-rating of all ten domains of information literacy and social responsibility after completing the program.

**Conclusions:**

This study showed that medical students are competent editors of Wikipedia and that their contributions improve both the quality of the articles and their own perceived information literacy. Additionally, editing medicine-related articles provides an opportunity to build students' social responsibility by improving content on an open platform that reaches millions each day.

## INTRODUCTION

We have all found ourselves staring at a Wikipedia page at some point in time. You probably questioned its validity, but did you ever stop to wonder who created the content and why? The validity of medically related Wikipedia articles is improving every day thanks to a growing group of physicians, soon-to-be physicians, and many other citizen scientists known as WikiProject Medicine. WikiProject Medicine is one of many WikiProjects that oversee the accuracy and validity of Wikipedia's content. A growing body of literature discusses the use of Wikipedia editing as a pedagogical tool in higher education [1]. An increasing number of editors are medical students thanks to Wiki Education (WikiEdu), the non profit that runs programs to support relationships between universities and Wikipedia. WikiEdu has overseen the use of Wikipedia as a teaching tool since 2013 [2]. Many undergraduate disciplines from chemistry to English have undertaken Wikipedia assignments to explore multiple aspects of communication, writing, publishing, and information literacy [3–6]. More recently, Wikipedia editing is increasingly being used in medical and allied health education to reinforce evidence-based medicine concepts through the search for and synthesis of the extant information resources [7–13].

Prior use of Wikipedia editing with first-year medical students has shown that it can improve their confidence in locating knowledge gaps, literature searching, group work, and selecting reliable sources [10]. Subsequent studies with third- and fourth-year medical students, and allied health students, cite an increase in students' self-perceived ability to practice evidence-based medicine and satisfaction with “giving back” to Wikipedia [7, 9]. As such, Wikipedia editing serves as a vehicle for social responsibility by allowing students to contribute to, improve, and expand health information read by millions [14]. Faulkner and McCurdy define social responsibility as “the state of being fit to be trusted, worthy of confidence, and dependable for the improvement of the health of society and its members ” [15]. They further state that a socially responsible person is someone who helps to promote a healthy society. A 2014 survey examined the motivations of Wikipedia editors who edited health-related articles [16]. They found many editors felt the responsibility to ensure that patients could access quality health information. The drive to provide access to information aligns well with the health profession and missions of libraries: to enhance health literacy and democratize information and knowledge [17, 18].

Medical education is increasingly incorporating Wikipedia as a teaching device. Still, there is a gap in the literature about how the editing activities can contribute to overall student learning outcomes and lifelong learning in medical students. For this study, we define lifelong learning in the context of information literacy skills, specifically, their ability to search and retrieve biomedical information, critically appraise the information they find, and their ability to apply it to patients and populations (in this case, to readers of Wikipedia) [19]. Most studies on Wikipedia in the classroom have been concentrated in fields outside of medicine [3–6], other sciences or allied health [11–13, 20, 21], or with more advanced third- and fourth-year medical students [7, 9]. Furthermore, most studies investigating information literacy outcomes through Wikipedia editing assignments are concentrated in fields outside of medicine [6, 12, 22–24]. This project sought to explore the impact of Wikipedia editing on information literacy skills, lifelong learning, and social responsibility of first-year medical students, with the intent to expand the use of Wikipedia editing activities in medical schools.

### Background

Azzam et al. at the University of California San Francisco (UCSF) are credited with creating the first medical school course to have medical students edit Wikipedia articles [7]. Their first course ran in 2013 as an elective for fourth-year medical students and has continued to varying degrees. Since UCSF's pioneering course, a number of other health profession schools, spanning four countries, have added some form of Wikipedia editing to their curriculum [8]. Based on the data provided by the Wiki Edu dashboard as of late 2021, the following health profession schools have taught with Wikipedia: Clarion and Edinboro Universities, DeSales University, Icahn School of Medicine at Mount Sinai, Medical University of South Carolina, Rush University Medical Center, Tel Aviv University, University of California, San Francisco School of Medicine, University of Central Florida College of Medicine, University of Hawai‘i John A. Burns School of Medicine, University of Illinois College of Medicine, University of Manitoba, University of Michigan Medical School, University of Texas Health Science Center, Texas College of Osteopathic Medicine, Vanderbilt University School of Medicine, Washington University School of Medicine, and Western Michigan University Homer Styker MD School of Medicine [25].

The medical education curriculum at the University of Hawai‘i at Mānoa, John A. Burns School of Medicine (JABSOM), uses a combination of problem-based learning (PBL) format and traditional didactic lectures, and together they form a unit. The first-year curriculum includes four units and is referred to as MD1, MD2, MD3, and MD4. The MD1 unit is titled “Health and Wellness” [26]. It serves as an introduction to medicine with a broad overview of the various themes students will encounter throughout their time in medical school.

Before teaching with Wikipedia, information literacy concepts were introduced via a one-shot library session during the first week of instruction for first-year medical students (MS1s). Skills practiced included analyzing clinical and other health-related questions, determining appropriate resources, and retrieving information from databases. MD1 unit coordinator, RK, wanted to expand current efforts to build information-related critical thinking skills. The librarian, MKH, wanted to explore ways of moving beyond the one-shot library session. In 2018, RK teamed up with MKH to test the feasibility of Wikipedia editing assignments with MS1s. After reviewing the Wikipedia editing exercises and lessons available through WikiEdu, RK and MKH felt that the lessons aligned well with the Information Literacy in Higher Education Framework presented by the Association of College and Research Libraries (ACRL) [27] and the JABSOM graduation objectives, informed by the standards set forth by the Liaison Committee on Medical Education (LCME) and evidence-based medicine (EBM) concepts [28].

### Purpose

The purpose of our project was to determine the feasibility and effectiveness of Wikipedia editing to improve information literacy and lifelong learning skills and to investigate aspects of social responsibility in first-year medical students.

The specific aims include

1. exploring the relationship and effect of Wikipedia-editing on perceived information literacy self-ratings with first-year medical students,
2. improving health-related Wikipedia articles as a form of social responsibility,
3. and exploring best methods for teaching with Wikipedia and medical students.

## METHODS

The Health Sciences Library (HSL) partnered with the Office of Medical Education (OME) of the John A. Burns School of Medicine (JABSOM) at the University of Hawai‘i at Mānoa to pilot the use of Wikipedia editing with first-year medical students over the course of two years. We consulted with Dr. Azzam to determine the feasibility of undertaking a Wikipedia editing project with first-year medical students at our institution. We also consulted Wiki Education content for teaching (instructional design) [29] and learning (WikiEdu tutorials) [30]. We mapped aspects of the ACRL framework into our survey instrument to assess outcomes [27]. We used the JABSOM graduation objectives and LCME guidelines for self-directed learning to inform our project's overall objectives. JABSOM's first graduation objective is that students will become lifelong learners: more specifically, that students will achieve this by “searching for and retrieving biomedical information, critically appraising this information, and applying it appropriately to patients and populations” [19].

**Figure 1:**
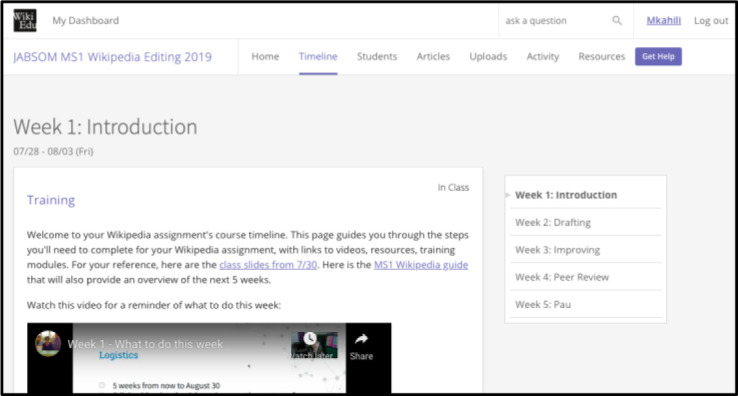
WikiEdu dashboard

The WikiEdu learning management system (LMS) was used to conduct the course. WikiEdu provides a series of lessons via the LMS that students work to complete. The tutorials range from Wikipedia editing policies to plagiarism to the essential technical skills for editing Wikipedia articles. Figure 1 shows a screenshot of the WikiEdu dashboard. Upon completion of the WikiEdu lessons, students selected a health-related Wikipedia article to edit. After the editing experience, feedback was collected to assess the students' overall outcomes and attitudes in relation to perceived information literacy skills via an anonymous retrospective pre-post survey. A summary of the program methods can be found in Table 1.

**Table 1:** Program summary

	2018 cohort	2019 cohort
**Participants**	72, worked individually	77, in 15 groups
**Training**	All asynchronous lessons, one video	Two in-person sessions, asynchronous lessons, six videos
**Frequency**	14 weeks	5 weeks
**Articles**	43	18
**Engagement**	SC mean before=41.0 SC mean after=45.8	SC mean before=42.5 SC mean after=57.8

### Program

#### Participants

Participants of this multiyear pilot project were first-year medical students at the JABSOM. Over two years, the project took place with the class entering in 2018 (2018 cohort) and the class entering in 2019 (2019 cohort). The pilot project and survey received approval from the UHM Institutional Review Board in 2018 (IRB #2018-00832). An Institutional Review Board modification was obtained in 2019 to include the 2019 cohort.

#### Training

In 2018, first-year medical students (MS1s) participated in Wikipedia editing activities entirely online and asynchronously. The course content was communicated through email and the WikiEdu LMS. Students completed the recommended tutorials from WikiEdu. In consultation with the course director, Dr. Richard Kasuya, the authors decided to engage and motivate students further in the following year. In 2019, the course used a combination of two required in-person sessions besides asynchronous learning through a variety of videos and tutorials embedded in the WikiEdu LMS. For the first iteration of the project in 2018, students worked independently. For the second iteration in 2019, students worked to edit their Wikipedia articles with their assigned PBL groups.

#### Frequency

In 2018, the project ran from August to November (fourteen weeks), which spanned the length of the MD1 unit and went partially into the next unit (MD2). WikiEdu recommends a course length from six to twelve weeks. The course's first iteration included breaks from editing to accommodate exams and the transition from MD1 to MD2. We thought the longer course length would lessen the pressure of the editing project and give students more time to get comfortable with the platform. After the project, we collected feedback from students to determine how we could improve the course delivery and assessed the overall quality of work the students had completed. We ultimately shortened the course to keep students more engaged in the course content. In 2019, the project ran from the end of July to September (five weeks).

#### Articles

The librarian (MKH) selected the students' articles by mapping existing articles to topics presented in their weekly PBL tutorial cases and then mapping the articles to the WikiProject Medicine grading scale [31]. WikiProject Medicine is a group of editors that oversee the medically related content in Wikipedia. They also assess articles for quality and provide classification to identify articles based on the quality and quantity of the content. At the bottom of the scale are articles with the least amount of content, classed as “Stub.” Articles starting development are classed as “Start.” Articles with a substantial amount of content but still missing key elements are classed as “C.” At the top of the scale are “GA” or good articles and “FA” or featured articles [31]. MKH selected articles rated Stub, Start, and C class with various degrees of importance from low to high. Choosing preexisting articles allowed students to work on Wikipedia articles that were aligned with what they were learning in their regular coursework. It also allowed us to assess the students' ability to affect the structural completeness of existing articles, thus providing an objective measure with which to assess student work.

#### Engagement

Following the initial run of Wikipedia editing in 2018, we had concerns with student engagement. We assessed engagement by assessing students' completion of course modules and the number and quality of edits they made to Wikipedia articles. Student feedback also indicated that an incentive could help encourage more engagement from the students. Given that the 2019 cohort would work in teams, we decided we would award a pizza party to the team with the highest structural completeness (SC) score, the measure by which we measured student engagement. Students were instructed on where to find the SC score in the WikiEdu LMS to measure the impact of the edits made to their articles.

### Assessments and measures

The survey instrument was an eleven-question, retrospective pre-post survey. Students rated their knowledge of various IL skills before the editing experience and then rated their knowledge again after the experience (Appendix A). The survey was created by consulting different measures targeting evidence-based practice, self-directed learning, and IL skills, focusing on items aligned with the ACRL framework [27, 32, 33]. We also consulted with EH, an experienced researcher in educational psychology, to further refine our scale.

The final scale (Appendix A) includes ten pre-post statements using a 5-point Likert scale, with 1 = strongly disagree, 5 = strongly agree, to assess the following: (1) did the Wikipedia editing project help to improve IL skills (how they find, use, cite information), and (2) did the project change their views on Wikipedia and their ability to contribute as a form of social responsibility? The items measured include (1) editing Wikipedia as a form of community service, (2) social responsibility, (3) ability to find resources, (4) ability to determine authority/credibility, (5) social nature of online information, (6) ability to find and use library resources, (7) Wikipedia as a valid and reliable source, (8) perception of evidence-based medicine, (9) ability to synthesize information, and (10) ability to cite.

Three additional questions were included to assess how the course aligned with the curriculum, what students liked about the course, what they would change, and any other comments they wanted to provide. The open-ended feedback helped us change the delivery of the program to improve it.

We also used the WikiEdu dashboard to collect and track data for the edits made to articles and their overall effect on SC (Appendix B). The SC data are calculated by the Objective Revision Evaluation Service (ORES) and use an algorithm that scores articles on completeness (e.g., headings present, content, and references) [32, 33]. The SC data allow us to compare the changes students make to the articles without critiquing the content itself, as we are not subject-matter experts. The SC data immediately informs users of positive changes (i.e., raised the overall SC score of the article), negative changes (i.e., lowered the SC score), or of unchanged scores. The SC scores also provided a measure of student engagement (i.e., the higher the SC score of the article, the more the student was engaged with the edits). In sum, collecting survey data addresses Aim 1. Analyzing SC directly addresses Aim 2 and Aim 3.

#### Analysis

Data for this project were analyzed using various tools, including proprietary analytic features of WikiEdu, Microsoft Excel 365, and IBM SPSS Statistics 26. Descriptive statistics were derived, and nonparametric tests were run to analyze program effects on pre- and post-program information literacy and social responsibility self-ratings given the occasional skewed distributions. Specifically, we used Wilcoxon signed-rank tests to determine differences between repeated measurements.

## RESULTS

As summarized in Table 1, 72 participants worked individually in the 2018 cohort, and 77 participants were working within 15 groups in the 2019 cohort. The 2018 cohort comprised 32 females and 40 males and ranged from 21 to 33 years old, with an average age of 24 at the program onset [34]. The 2019 cohort was composed of 39 females and 38 males and ranged from 21 to 45 years old, with an average age of 24 [35]. The study aims not to compare the two cohorts, but we performed exploratory analyses of the data to gauge if the changes to the delivery of the course were effective or not.

### Information literacy, lifelong learning, and social responsibility

Fifty-seven (79%) participants in the 2018 cohort and 49 (64%) participants in the 2019 cohort completed the retrospective information literacy survey. A summary of the findings can be seen in Table 2. In both cohorts, respondents showed statistically significant increases (*p*<.05) in self-ratings of all ten domains of information literacy and social responsibility after completing the program.

**Table 2:** Information literacy self-ratings

Q	2018 cohort (N=57)								2019 cohort (N=49)							
	Pre			Post			Test		Pre			Post			Test	
	Mean	SD	Med.	Mean	SD	Med.	Z	*p*	Mean	SD	Med.	Mean	SD	Med.	Z	*p*
1	3.0	1.1	3.0	3.8	1.1	4.0	−4.635	<.001	2.8	1.0	3.0	4.0	0.7	4.0	−5.248	<.001
2	2.5	1.0	2.0	3.0	1.2	3.0	−4.199	<.001	2.1	1.0	2.0	3.4	0.8	3.0	−5.493	<.001
3	3.8	1.0	4.0	4.1	0.8	4.0	−3.700	<.001	3.3	1.3	4.0	4.3	0.8	4.0	−4.627	<.001
4	3.7	1.1	4.0	4.2	0.7	4.0	−3.955	<.001	3.5	1.1	4.0	4.2	0.7	4.0	−4.582	<.001
5	3.6	1.1	4.0	4.2	0.8	4.0	−4.715	<.001	3.3	1.2	3.0	4.1	0.8	4.0	−5.002	<.001
6	4.0	1.0	4.0	4.3	1.0	5.0	−2.859	.004	4.0	1.2	4.0	4.5	0.9	5.0	−3.877	<.001
7	3.1	1.1	3.0	3.5	1.0	4.0	−4.030	<.001	2.4	1.1	2.0	3.2	1.1	3.0	−4.971	<.001
8	4.6	0.7	5.0	4.7	0.6	5.0	−2.060	.039	4.4	0.9	5.0	4.8	0.4	5.0	−3.354	<.001
9	4.0	0.9	4.0	4.4	0.6	4.0	−3.753	<.001	3.9	0.9	4.0	4.4	0.6	4.0	−4.134	<.001
10	4.0	0.9	4.0	4.3	0.7	4.0	−3.358	<.001	3.8	0.9	4.0	4.3	0.6	4.0	−4.300	<.001

* SD = standard deviation, Med. = median, Wilcoxon text

** 1 = Editing Wikipedia as a form of community service, 2 = Social Responsibility, 3 = Ability to find resources, 4 = Ability to determine authority/credibility, 5 = Social nature of online information, 6 = Find and use library resources, 7 = Wikipedia as valid and reliable source, 8 = EBM, 9 = Ability to synthesize information, 10 = How to cite

**Figure 2:**
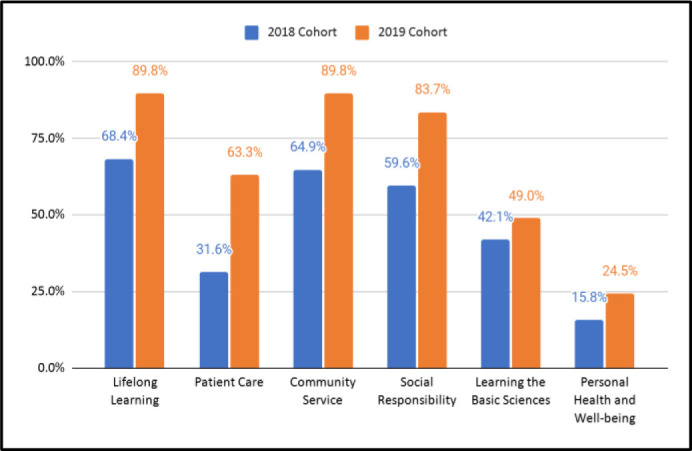
Percent of cohort demonstrating commitment or contribution through Wikipedia editing

Note. Significant (*p*<.05) changes from the 2018 to 2019 cohort in commitment to lifelong learning, patient care, community service, and social responsibility.

Note. Item 11: *I believe that Wikipedia editing is one way of demonstrating my commitment/contribution to …*

Figure 2 shows curriculum outcomes that the Wikipedia editing experience targeted. In the first iteration of the program involving the 2018 cohort, more than half of students endorsed a commitment to lifelong learning (68.4%), community service (64.9%), and social responsibility (59.6%). The 2019 cohort overwhelmingly identified with a commitment to lifelong learning (89.8%), community service (89.8%), social responsibility (83.7%), and patient care (63.3%). This cohort endorsed the following types of contribution in greater frequencies than the prior cohort: lifelong learning (χ2=7.086, *p*=.008), patient care (χ2=10.643, *p*=.001), community service (χ2=9.053, *p*=.003), and social responsibility (χ2=7.349, *p*=.007). The 2019 cohort did endorse increased commitment to learning the basic sciences (χ2=.0503, *p*=.478) or personal health and well-being (χ2=1.255, *p*=.263).

### Program delivery

#### Editing

Table 3 summarizes the contributions by cohort. The 2018 cohort provided 613 edits over 51 articles, changed roughly 14,300 words, and added 234 references. The 2019 cohort collectively made 830 edits to 18 articles, as they worked in groups, unlike the previous cohort. Roughly 18,600 words were changed, and 254 references were added.

**Table 3:** Summary of Wikipedia edits

	Students	Articles	Edits	Words	References	Views
2018	72	51	613	14,300	234	2,780,000
2019	77	18	830	18,600	254	450,000

Note. Views are current as of March 2021.

#### Article improvement

We tracked article structural completeness using the WikiEdu dashboard to measure overall quality improvement before and after the program (Appendix B). The histograms presented in Appendix B illustrate mean scores of article structural completeness on the x-axis and frequency or count of articles with that structural completeness score on the y-axis. In the 2018 cohort, mean structural completeness scores improved from a mean of 41.2 and standard deviation of 14.7 to a mean of 43.9 and standard deviation of 13.1 in 43 edited articles, indicating a statistically significant improvement (t=2.994, *p*=.005). In the following cohort, scores improved from a mean of 43.3 and standard deviation of 14.0 to a mean of 58.9 and standard deviation of 11.0 over 18 edited articles, showing a statistically significant improvement (t=4.712, *p*<.001). The 2019 cohort improved articles by a mean of 15.6 and a standard deviation of 14.0. The 2018 cohort improved articles by only a mean of 2.7 and a standard deviation of 6.0, indicating a significant difference in the cohorts (t=3.756, *p*=.001). In the 2018 cohort, 28 (65%) articles were improved while 8 (19%) articles were made worse. Sixteen (89%) articles were improved in the following cohort, and none were made worse.

Table 4 details the most improved articles by cohort. The most improved articles included “Trauma team” (43.7), “Complication (medicine)” (36.1), “Respiratory examination” (31.1), and “Coronary arteries” (29.7), all of which were Start-class articles and edited by the 2019 cohort. Only two of the ten most-improved articles were edited by the 2018 cohort: “Hypopigmentation” (20.1, Stub-class) and “Dermatophyte” (19.7, Start-class).

**Table 4:** Most improved articles by cohort

2018 cohort			2019 cohort		
Article	Class	Structural completeness score improvement	Article	Class	Structural completeness score improvement
Hypopigmentation	Stub	20.1	Trauma team	S	43.7
Dermatophyte	S	19.7	Complication (medicine)	S	36.1
Abdominal examination	S	14.1	Respiratory examination	S	31.1
Parasitic disease	S	13.9	Coronary arteries	S	29.7
Rapid influenza diagnostic test	Not rated	12.8	Vital signs	C	24.4
Thrombus	S	9.4	Coccus	S	23.8
SOAP note	C	8.3	Cardiac monitoring	S	19.6
Hawaii Department of Health	Stub	7.6	The Queen's Medical Center	S	19.5
Leprosy stigma	S	5.1	Metabolic acidosis	C	18.5
Vitamin B3	Stub	4.8	SOAP note	C	18.3

Note. Stub = least content; S = Start, some content; C = Substantial but missing important content

## DISCUSSION

### Summary

We attribute the success of the project to several factors: the ability to work with the librarians more compared to the previous one-shot sessions, the ability to communicate information literacy concepts and then have students apply them in a meaningful way, the improved dashboard content, the compressed timeline, and the students' ability to work in groups. This study shows that Wikipedia editing activities increased first-year medical students' perceived information literacy skills. Similar to previous studies [6, 10, 12, 23, 24, 36], Wikipedia editing can improve information literacy skills (e.g., finding information, using library databases, evaluating information, and citing). Specifically, this effort lines up with findings by Murray et al. Wikipedia editing can be used to teach evidence-based medicine lessons (specifically, searching and selecting information) with first-year medical students [10].

In terms of social responsibility, it also confirms the work of other studies that have examined the motivations of Wikipedia editors [16]. The medical students were able to connect editing to improve Wikipedia health-related articles to social responsibility and community service. As an information resource that anyone can edit, medical professionals have the responsibility to ensure that the content is accurate. In general, the number of WikiProject Medicine editors is declining [37]. Introducing students to Wikipedia early in medical education will hopefully motivate them to contribute to the resource once they are professionals.

Regarding best teaching methods, the changes made to the delivery of the 2019 program showed improved student outcomes. We used feedback from the first iteration to improve course engagement by offering an incentive to students for participation, providing more guidance through videos and more face-to-face time. Multiple options for navigating the course were also offered. The course timeline was compressed to five weeks to maintain course momentum. The retrospective survey achieved statistical significance on all ten survey questions. A comparison of changes in the overall structural completeness of the edited articles shows that the second iteration of the course achieved a higher mean. Our success with group editing differs from other outcomes seen in previous studies [24]. We attribute this to our students' graduate-level standing, their understanding and acceptance of the PBL curriculum, and the understanding that health care is ultimately team-based.

There are other implications worth mentioning. The average Wikipedia editor is male, English speaking, and from North America, and gender bias in Wikipedia has been well documented [37–39]. At JABSOM, the majority of the class are Hawai‘i state residents, with Hawai‘i state demographics as of 2019 being 37.6% Asian (alone), 25.5% White (alone), 24.2% two or more races, and 10.1% Native Hawaiian and other Pacific Islander alone [40]. The 2018 cohort was comprised of thirty-two females and forty males [34]. The 2019 cohort was comprised of thirty-nine females and thirty-eight females [35]. Though not our intention, this project was successful in diversifying the Wikipedia editor pool.

### Lessons learned

The initial run of the project was deemed a success based on survey data measuring students' self-reported perceived information literacy skills. After consulting with the course director, the decision was made to rerun the project in 2019. After adjusting how the course was delivered, we saw an even more significant improvement in students' perceived information literacy skills and more remarkable changes to the Wikipedia article structure than in 2018.

The authors attribute their specific, strategic changes to the course format for improvement in the second iteration. These are (1) shorter editing time to maintain course momentum, (2) in-person sessions to provide more guidance and establish rapport between librarians and students, (3) students working with their PBL group as a team, just as they do during PBL group sessions, and (4) students were motivated by a pizza party for the team with the greatest overall structural change [41]. In sum, we saw better outcomes with medical students editing as a team. This change was more reflective of their PBL curriculum and expectations as budding health care professionals.

In 2020 and 2021, the program was slated to run again but was ultimately canceled, given the significant changes to education delivery due to the COVID-19 pandemic. The University of Hawai‘i moved all classes online in March 2020. Since then, the curriculum and students alike have adjusted to online learning. With students now more accustomed to online learning, we believe the Wikipedia editing experience could be delivered online again with a combination of asynchronous and synchronous learning opportunities via video conferencing applications such as Zoom. Using Zoom breakout rooms could allow us to recreate some of the camaraderie students experienced with in-person learning.

### Strengths and limitations

There were several strengths and limitations in our project. One major strength was having the agreement and support of medical education faculty at the JABSOM. The willingness of medical education faculty to partner with the library allowed us to work with students in a way we never had before. Another strength was engaging student participation. Not only were students new to editing Wikipedia, but they were also new to medical school and dealing with a lot of uncertainty at the time. Their willingness to participate helped make this project a success. Finally, we did not rely on a post-only evaluation design; instead, we used a pre-post design based on the retrospective assessment of the medical students. The retrospective pre-post survey design allowed us to avoid the possibility of students overestimating or underestimating their knowledge (i.e., you don't know what you don't know).

There are limitations to the present study. The survey has not yet been psychometrically validated. In terms of evaluation design, the retrospective pre-post design, while superior to a post-only design, may still have involved biases (e.g., recall, social desirability) and was susceptible to internal validity threats (e.g., history, maturation). Although the students were simultaneously participating in regular PBL sessions and lectures, three of the ten items were specific to Wikipedia. However, construct and items were tapped into that had previous empirical support, and given the statistically significant results, the items were at least reliable. Our study approach most closely resembles participatory action research, a methodology frequently used in education for improving the delivery of a course through research, assessment, and reflection [37, 38]. This meant changing course delivery from the 2018 and 2019 cohorts (second cohort had different methods, training, incentive, group). Although the response rates of 79% and 64% for the 2018 and 2019 cohorts, respectively, were moderately high, we do not know how those who took part were similar or different from those who did not participate. Finally, SC is calculated through an algorithm that we did not have complete access to or control of. A final limitation, we did not qualitatively analyze students' open-ended feedback, which may have correlated with the SC score for measuring engagement.

## CONCLUSION

Piloting this project helps to normalize the use of Wikipedia as a teaching tool in medical schools. This project also shows the feasibility of teaching with Wikipedia in medical education curriculums. The second iteration of the project was also a better reflection of the Wikipedia editing experience—working in teams collectively to improve the articles. Anecdotally, the relationship between the library and the students was improved; being embedded in the first-year curriculum allowed us to work with students in new and meaningful ways. Overall, there are valuable lessons and skills for students to learn through the Wikipedia editing experience. Wikipedia editing is a novel method to teach students information literacy that will impact their academic and professional lives. As the students make their way through medical school, their skills in Wikipedia editing will become increasingly important. They will need to know how to find, use, interpret, and synthesize valid information to ensure effective patient care. This project also contributes to the larger mission of Wikipedia: to increase access to information.

## Data Availability

The datasets analyzed for this study are available at https://scholarspace.manoa.hawaii.edu/handle/10125/75664.

## SUPPLEMENTAL FILES

**Appendix A:** Self-rated information literacy survey items

**Appendix B:** Change in structural completeness of articles edited
