# Supplementary material for: Library instruction and Wikipedia: investigating students' perceived information literacy, lifelong learning, and social responsibility through Wikipedia editing
Source: J Med Libr Assoc. 2022 Apr 1;110(2):174–84. doi: 10.5195/jmla.2022.1291 (PMC9014945; doi:10.5195/jmla.2022.1291)
Supplement: Supplementary file 1 — Appendix A: Self-rated information literacy survey items [file jmla-110-2-174-s01.pdf]

Appendix A

Self-rated information literacy survey items

Statements 1–10 are rated retrospectively before and after on a scale of 1–5 (1=strongly disagree, 5=strongly agree)

1. I view Wikipedia editing as a form of community service.
2. I feel a sense of social responsibility to contribute to Wikipedia.
3. I know how to find resources for my assignments.**
4. I know how to determine the authority and credibility of sources.*
5. I understand the increasingly social nature of online information and how sources develop over time.*
6. I use the library resources to access biomedical/scholarly information.***
7. I consider Wikipedia to be a valid and reliable resource for medical learning.
8. I believe the ability to find evidence-based information is vital to the practice of medicine.***
9. I know how to synthesize information gathered from multiple sources.*
10. I know how to give credit through proper citation.*
11. I believe that Wikipedia editing is one way of demonstrating my commitment/contribution to:
    1. Lifelong Learning
    2. Patient Care
    3. Community Service
    4. Social Responsibility
    5. Learning the Basic Sciences
    6. Personal Health and Well-being
12. What were the attributes of the Wikipedia Editing Project that could be improved upon, and for each, what could be done to improve the course?
13. What were the positive aspects of the Wikipedia Editing Project?
14. Any other comments?

Note: Survey items derived from:
* Framework for Information Literacy for Higher Education. American Library Association. http://www.ala.org/acrl/standards/ilframework (Accessed June 28, 2021). Document ID: b910a6c4-6c8a-0d44-7dbc-a5dcbd509e3f
** Cheng S-F, Kuo C-L, Lin K-C, Lee-Hsieh J. Development and preliminary testing of a self-rating instrument to measure self-directed learning ability of nursing students. Int J Nurs Stud. 2010 Sep;47(9):1152–8.
*** Long JD, Gannaway P, Ford C, Doumit R, Zeeni N, Sukkarieh-Haraty O, Milane A, Byers B, Harrison L, Hatch D, Brown J, Proper S, White P, Song H. Effectiveness of a technology-based

intervention to teach evidence-based practice: the EBR tool. Worldviews Evid Based Nurs. 2016 Feb;13(1):59–65.
