# Supplementary material for: Library instruction and Wikipedia: investigating students' perceived information literacy, lifelong learning, and social responsibility through Wikipedia editing
Source: J Med Libr Assoc. 2022 Apr 1;110(2):174–84. doi: 10.5195/jmla.2022.1291 (PMC9014945; doi:10.5195/jmla.2022.1291)
Supplement: Supplementary file 2 — Appendix B: Change in structural completeness of articles edited [file jmla-110-2-174-s02.pdf]

Appendix B

Change in structural completeness of articles edited

2018 Cohort

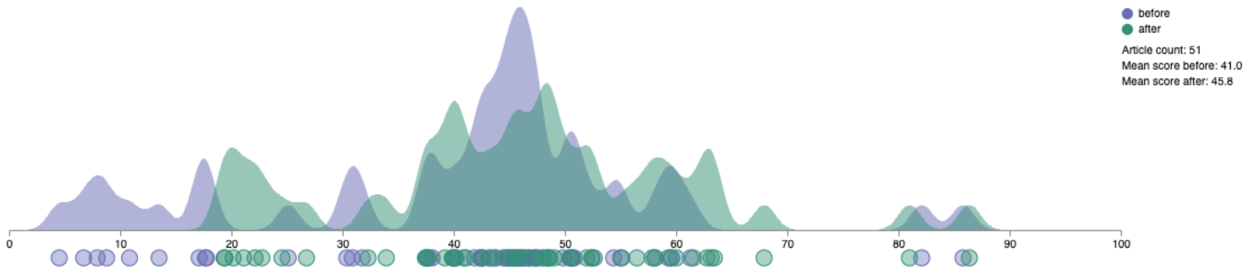

2019 Cohort

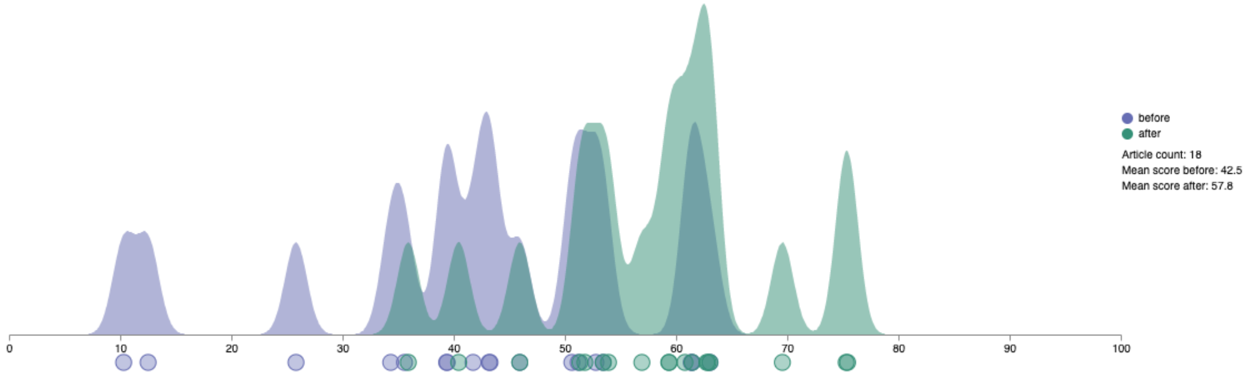

Note: Data presented as computed by the machine learning algorithm, ORES (https://www.mediawiki.org/wiki/ORES/FAQ). The histograms illustrate mean scores of article structural completeness on the x-axis and frequency or count of articles with that structural completeness score on the y-axis.
